# Extracorporeal life support in pediatric cardiac dysfunction

**DOI:** 10.1186/1749-8090-5-112

**Published:** 2010-11-17

**Authors:** Kasim O Coskun, Sinan T Coskun, Aron F Popov, Jose Hinz, Mahmoud El-Arousy, Jan D Schmitto, Deniz Kececioglu, Reiner Koerfer

**Affiliations:** 1 Department of Thoracic and Cardiovascular Surgery University of Göttingen, Göttingen, Germany; 2 Department of Cardiovascular Surgery, Heart and Diabetes Centre North-Rhine Westphalia, Ruhr-University of Bochum, Bad Oeynhausen, Germany; 3 Department of Anaesthesiology, Emergency and Intensive Care Medicine, University of Göttingen, Göttingen, Germany; 4 Department of Pediatric Cardiology, Heart and Diabetes Centre North-Rhine Westphalia, Ruhr-University of Bochum, Bad Oeynhausen, Germany

## Abstract

**Background:**

Low cardiac output (LCO) after corrective surgery remains a serious complication in pediatric congenital heart diseases (CHD). In the case of refractory LCO, extra corporeal life support (ECLS) extra corporeal membrane oxygenation (ECMO) or ventricle assist devices (VAD) is the final therapeutic option. In the present study we have reviewed the outcomes of pediatric patients after corrective surgery necessitating ECLS and compared outcomes with pediatric patients necessitating ECLS because of dilatated cardiomyopathy (DCM).

**Methods:**

A retrospective single-centre cohort study was evaluated in pediatric patients, between 1991 and 2008, that required ECLS. A total of 48 patients received ECLS, of which 23 were male and 25 female. The indications for ECLS included CHD in 32 patients and DCM in 16 patients.

**Results:**

The mean age was 1.2 ± 3.9 years for CHD patients and 10.4 ± 5.8 years for DCM patients. Twenty-six patients received ECMO and 22 patients received VAD. A total of 15 patients out of 48 survived, 8 were discharged after myocardial recovery and 7 were discharged after successful heart transplantation. The overall mortality in patients with extracorporeal life support was 68%.

**Conclusion:**

Although the use of ECLS shows a significantly high mortality rate it remains the ultimate chance for children. For better results, ECLS should be initiated in the operating room or shortly thereafter. Bridge to heart transplantation should be considered if there is no improvement in cardiac function to avoid irreversible multiorgan failure (MFO).

## Introduction

Despite technical improvements in congenital heart surgery, mortality as a result of cardiac dysfunction after corrective surgery remains a serious problem. A total of 1 to 5% of these patients will require some form of mechanical support [1-3]. In addition, children with dilatated cardiomyopathy (DCM) may also require extracorporeal life support (ECLS) due to multiorgan dysfunction if conservative medical treatment is inadequate.

In this retrospective single center analyzes we present our experience with both extra corporeal membrane oxygenation (ECMO) and ventricle assist device (VAD) for pediatric patients requiring ECLS at our institution. We reviewed the outcomes of pediatric patients necessitating ECLS after corrective surgery and compared outcomes with pediatric patients necessitating ECLS because of DCM. Our aim is to report the prognosis of children undergoing ECLS and to compare the outcomes of the two main diseases associated with high mortality even in canters with ECLS possibilities.

## Materials and methods

A total of 48 patients received ECLS, of which 23 were male and 25 female. The indications for ECLS included CHD in 32 cases and DCM in 16 patients. The mean age was 1.2 ± 3.9 years for CHD patients and 10.4 ± 5.8 years for DCM patients. Twenty-six patients received ECMO; 22 patients in CHD group vs. 4 patients in DCM group and 22 patients received VAD; 10 patients in CHD group vs. 12 patients in DCM group.

The preoperative diagnoses in CHD group included: 14 transposition of the great vessels, 1 Bland-White-Garland syndrome, 6 tetralogy of Fallot, 2 hypoplasia of the aortic arch, 2 total anomalous pulmonary vein connection, 4 univentricular heart and 3 ventricular septal defect. Patient characteristics are given in Table 1. Causes of DCM are not reported in this study since myocardial biopsies was not available in all patients. Indication for an ECLS is achieved after failing attempts weaning off from cardiopulmonary bypass (CPB) under pharmacological support or clinical deterioration and necessitating resuscitation.

**Table 1:** Clinical characteristics

Characteristics	DCM patients (n = 16)	Congenital patients (n = 32)	p-value
Age at surgery (years)	10.4 ± 5.8	1.2 ± 3.9	0.007
Weight	43 ± 29.2	6.9 ± 13.1	0.0001
Male/female	6/10	17/15	0.30
CPR before ECLS	1 (6.25%)	9 (28.1%)	0.078
CVVH	1 (6.25%)	11 (34.4%)	0.03
HTX	6 (37.5%)	1 (3.13%)	0.001
Bleeding with ECLS	2 (12.5%)	12 (37.5%)	0.07
Pump head exchange	0	5 (15.6%)	0.09
Duration of ECLS (days)	48.5 ± 78.5	7.8 ± 12.1	0.001
Survival after ECLS (days)	563.4 ± 929.4	464.2 ± 848.6	0.58
ECMO Assist device(LVAD/RVAD/BVAD)	4 (25%) 12 (75%)	22 (69%) 10 (31%)	0.004
Mortality	12 (75%)	21 (65.6%)	0.50

CPR: cardiopulmonary resuscitation, CVVH: continuous venous-venous hemofiltration, HTX: heart transplantation, ECLS: extracorporeal life support, ECMO: extracorporeal membrane oxygenation, LVAD: left ventricle assist device, RVAD: right ventricle assist device, BVAD: biventricular assist device

The aim of ECLS initiation was:

• The maintance of systemic circulation

• Recovery of multiple organ failure

• Bridge to transplantation

The patients received an ECLS support in case of:

• Inability to wean from CPB in the operation room

• Clinical deterioration: Despite optimal pharmacological support

• Low output syndrome,

• Mean arterial pressure <60 mmHg,

• Ejection fraction <25%

• Cardiac index <2 l/min/kg

• Diuresis <1 ml/min/kg

• Central venous pressure >15 mmHg

• Left atrial pressure > 18 mmHg

Cannulation of ECLS was performed either in the operating room or in the intensive care unit. The patient was given 30-100 units/kg of heparin, with ECMO; the activated clotting time is usually maintained between 170 and 200 seconds compared to 140-160 seconds in children on VAD. On institution of ECMO, inotropic support was weaned to minimal levels to keep mean arterial blood pressures at 50 mm Hg. Flow rates were maintained depending on hemodynamic situation until the SVO2 was 75%. The mean blood pressure range for neonates on ECMO is 40-65 mm Hg. Normothermia was maintained in all patients. In VAD group anticoagulation was started 24 hours after implantation after chest tubes removal Warfarin sodium (Coumadin; Bristol-Myer Squibb Company, Princeton, NJ) was initiated to maintain an INR value of 2.5-3.5. The used devices were MEDOS HIA-VAD (MEDOS Medizintechnik GmbH, Stollberg, Germany) - a pneumatically actuated blood pump, Thoratec paracorporeal pneumatic VAD (Thoratec Corp, Plesanton, CA), CardioWest total artificial heart (TAH) (SynCardia Systems, Tucson, AZ, USA). Novacor LVAD (Baxter, Oakland, NJ), ECMO with an oxygenator (Carmeda Maxima; Medtronic, Düsseldorf, Germany), and centrifugal pump (Biomedicus; Medtronic). None of the patients had an intra-aortic balloncounter pulsation (IABP).

Echocardiography is used to evaluate the ventricular function after 24 hours. Our criteria to initiate a left VAD-system included: good right ventricular contraction, adequate oxygenation and right atrial (RA) pressure <12 mmHg. Reducing the flow rate should allow to maintain adequate left ventricle (LV) ejection with left atrial (LA) pressure of 8-10 mmHg. If right ventricular (RV) function was poor and if patients did not show improvement, they should be converted to an ECMO for better oxygenation or they received an extra support like biventricular assist device (BVAD) or right ventricular assist device (RVAD. In cases of right ventricular failure, therapeutic measures included volume followed by nitric oxide inhalation, inotropic agents, phosphodiesterase type III inhibitors and prostaglandin depending on hemodynamic situation for each patient individually [4].

*The Indications for BVAD are*

• MOF

• cardiac failure

• CVP >20 mmHg

• PAP/CVP gradient < 4 mmHg

• PVR >500 dyn/sec/cm-5

### Statistical evaluation

**Figure 1:**
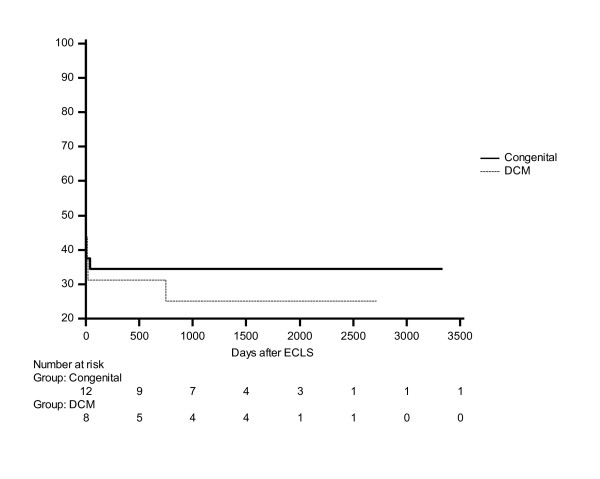
**Cumulative survival analysis of both groups as Kaplan-Meier survival function**. (Logrank test: p = 0.65).

Statistical analysis was performed using commercially available statistics software (Statistica 5.1., StatSoft Inc., Tulsa, OK, USA). Statistics were performed using Mann-Whitney U test for nonparametric continuous data and x2-test. Patient survival rates were calculated according to the Kaplan-Meier life table method (Figure 1). Statistical difference was considered at p < 0.05.

## Results

There was a significant difference in age and weight between the groups. DCM patients were older (10.4 ± 5.8 vs. 1.2 ± 3.9; p = 0.007) and had more body weight (43 ± 29.2 vs. 6.9 ± 13.1; p = 0.0001) than congenital patients. Gender achieved no statistically difference between the groups. Acute renal failure, which had to treated with continuous veno venous hemofiltration (CVVH), were more frequent in congenital patients than in DCM patients (11 vs. 1; p = 0.03). In DCM patients were more heart transplantations performed than in congenital patients (6 vs. 1; p = 0.001). Furthermore, the duration of ECLS was significant longer in DCM patients than in congenital patients (48.5 ± 78.5 vs. 7.8 ± 12.1; p = 0.001). In DCM patients more assist device (LVAD/RVAD/BVAD) were used and less ECMO than in congenital patients (p = 0.004). There were no statistically significances observed in bleeding with ECLS, pump head exchange, and survival after ECLS.

The mortality was quite uniform across the groups and was analyzed with Logrank test (p = 0.65), as shown in Figure 1.

## Discussion

The general indication for ECLS is inadequate organ perfusion due to ventricular dysfunction. The criteria and guidelines for choosing correct type of ECLS remains variable and controversial since heterogeneous group of patients are effected whose outcome is greatly influenced by multiple demographic, anatomic, clinical, surgical and post operative variables. The selection of device for the individual patients must be taken in consideration. The decision to implant an ECLS is based not only on the hemodynamic situation, but also the status of organ function. We must take into consideration that many post surgical problems are likely attributable to the preoperative condition of the patient, thus it is imperative to decide on possible implantation of a device before multi organ failure occurs. ECLS plays an important role as an alternative to support patients who might not otherwise survive - patients with intractable heart failure, low output or consequent MOF.

Complex CHD corrective operations mostly need postoperative support some because of late presentation and subsequent left ventricular failure, some because of residual lesions, coronary ischemia, poor myocardial protection and technical problems. All those factors increase mortality and need of ECLS. ECMO is more widely used in the pediatric population for short-term support and biventricular dysfunction Some authors confirm that ECMO is superior to VAD in CHD corrective surgery with cyanotic lesions with cardiac shunts, pulmonary hypertension and respiratory failure, whereas VAD systems are often indicated for univenticular failure - for mid to long-term assistance [5,6].

IABP is not adequate for these critical situations; the optimal approach to preserve end organ function is instituting VAD or ECMO support before extended periods of LCO, arrhythmia or cardiac arrest.

Renal failure is a predictor of high mortality in VAD patients. Rapidly deteriorating patients should lead physicians to take an aggressive stance toward implantation of ECLS [2,7,8].

The overall hospital survival for pediatric patients managed with ECLS ranges between 38% and 53%, with long-term survival of infants and children at our institution of 31% (similar to that reported rates above) [9-17]. Nevertheless, the use of ECLS has a significantly high mortality rate associated with cardiopulmonary failure, multi-organ dysfunction, neurological dysfunction, deficiency of coagulation factors and mechanical factors [18]. It should be strongly considered that the mortality in those children ranged up to 90% if they do not receive any supports [2].

The mechanical complications have an incidence of 27%, including: oxygenator failure, clots in the circuit, pump malfunction, and presence of air in the circuit. These complications correspond to long run times [19]. Moreover, ECMO and centrifugal pumps require high levels of anticoagulation, which increases the risk of bleeding. With ECMO, the activated clotting time is usually maintained between 170 and 200 seconds compared to 140-160 seconds in children on VAD. Therefore, bleeding is the major complication of ECMO, and the most common sites for bleeding are cannulation and surgical sites [20].

However, we found that re-exploration for bleeding did not influence the overall clinical outcome [13].

The importance of brain protection and early identification of cerebral injury indicates the importance of early ECLS initiation. Neurological events in ECLS vary from 11 to 45% (19). Decision on bridging to heart transplantation, weaning off or device withdrawal depends on evaluation of neurological events The ELSO registry data indicated that cardiopulmonary resuscitation (CPR) before the initiation of ECMO does not have a negative impact on outcome, contrarily CPR in the pre-ECLS period improves survival rates of up to 60% among neonates [19].

The estimated weaning rate from ECMO is 43% [21] and poor prognosis has been reported in patients treated by ECMO for longer than 8 or 10 days [17,20,22].

Unfortunately high mortality rates are expected in DCM patients because of lack of heart transplantation opportunities, delay in referral for heart transplantation and subsequent development of MOF. In case of heart transplantation possibilities the literature shows an encouraging survival rate over 44% in patients bridged to cardiac transplantation and a 12 month survival of 62% to 88% [17,19,23-26]. In our experience, the application of mechanical circulatory support has also been useful as a bridge to heart transplantation with a survival rate of 71%, which correlates with our previous paper reviewing the outcome of pediatric heart recipients with CHD and DCM [26].

It is important to note that this study had some limitations. Although we reviewed a relatively large number of patients between 1991 an 2008, this remains a retrospective study. A heterogeneous group of patients are affected whose outcome is greatly influenced by multiple demographic, anatomic, clinical, surgical and post operative variables. There were data elements, i.e. lactate level, cardiac biopsy results and echocardiography not available for the entire cohort. Furthermore, a complete neurological evaluation was not always available, thus embolic or ischemic cerebrovascular events were not analyzed.

## Conclusion

ECMO and VAD remains the mainstay of mechanical circulatory support for children. The progress and development of ECLS is on-going and may possibly, in the near future, become a more effective and rapid support treatment option. ECMO, rather than VAD, may become the first line of treatment of choice - with faster initiation and fewer complications. For better results, ECLS should be initiated in the operating room or shortly thereafter to avoid prolonged hypoperfusion and a catastrophic cardiac arrest. However, if there is no improvement in cardiac function, than patients should be bridged to VAD or heart transplantation.

## Competing interests

The authors declare that they have no competing interests.

## Authors' contributions

OC, SC, and ME and had helped with surgical techniques, performed data, analysis, statistics, graphics, and wrote the paper. AP and JS and helped with data interpretation and helped to draft the manuscript. DK and RK co-wrote the manuscript and added important comments to the paper. All authors read and approved the final manuscript.
